# PRP Augmentation for ACL Reconstruction

**DOI:** 10.1155/2015/371746

**Published:** 2015-05-05

**Authors:** Luca Andriolo, Berardo Di Matteo, Elizaveta Kon, Giuseppe Filardo, Giulia Venieri, Maurilio Marcacci

**Affiliations:** ^1^II Orthopaedic and Traumatology Clinic, Biomechanics and Technology Innovation Laboratory, Rizzoli Orthopaedic Institute, Via di Barbiano No. 1/10, 40136 Bologna, Italy; ^2^Nano-Biotechnology Laboratory, Rizzoli Orthopaedic Institute, Via di Barbiano No. 1/10, 40136 Bologna, Italy

## Abstract

Current research is investigating new methods to enhance tissue healing to speed up recovery time and decrease the risk of failure in Anterior Cruciate Ligament (ACL) reconstructive surgery. Biological augmentation is one of the most exploited strategies, in particular the application of Platelet Rich Plasma (PRP). Aim of the present paper is to systematically review all the preclinical and clinical papers dealing with the application of PRP as a biological enhancer during ACL reconstructive surgery. Thirty-two studies were included in the present review. The analysis of the preclinical evidence revealed that PRP was able to improve the healing potential of the tendinous graft both in terms of histological and biomechanical performance. Looking at the available clinical evidence, results were not univocal. PRP administration proved to be a safe procedure and there were some evidences that it could favor the donor site healing in case of ACL reconstruction with patellar tendon graft and positively contribute to graft maturation over time, whereas the majority of the papers did not show beneficial effects in terms of bony tunnels/graft area integration. Furthermore, PRP augmentation did not provide superior functional results at short term evaluation.

## 1. Introduction

Anterior cruciate ligament (ACL) tears are among the most common sport-related injuries and therefore ACL reconstructive surgery is one the most frequently performed procedures in the field of sport medicine [1]. Epidemiological data reveal that the majority of patients are young and sport-active, with high expectations in terms of functional recovery and return to sport [2]. There is a flourishing literature concerning ACL reconstructive procedures: several different techniques have been documented over the years and, despite overall good clinical outcomes reported at mid/long-term followup, ACL is still on the edge of current clinical and preclinical research [3].

ACL surgery (and the related research) is a classic example of integration between biomechanics and biology: the progress made in recent decades can be attributed to the big steps forward in the knowledge of both the mechanical and biological properties of ACL and its healing process [4–8]. Although almost all available techniques can provide satisfactory results, ACL reconstruction is not a “100%-success” procedure: not all patients are able to regain their previous sport activity level and many factors could influence the clinical outcome, such as the type of graft used and the pre-op. knee laxity [2, 9]. This is a major concern since ACL injured patients can be a very demanding category, especially professional sport players who also need to return to the playing field as soon as possible. Current research is therefore investigating novel strategies to enhance ACL healing, to reduce the failure rate and accelerate recovery time. Among the different available options, biological augmentation is the most sought after approach and recently platelet rich plasma (PRP), which is an autologous blood derivative, largely applied in orthopaedic practice especially in the treatment of degenerative cartilage and tendon lesions, is gaining increasing interest [10–12]. PRP is a source of several growth factors (GFs) and other bioactive molecules that might promote tissue healing and regulate joint homeostasis [13, 14]. It is an easily available product obtained directly from the venous blood of the patient and its use is allowed also in athletes, since antidoping regulations do not consider this blood derivative as a banned substance [15]. Furthermore, it is a versatile product since it can be prepared and used directly in the operating theatre, through intra-articular injections or in the shape of a membrane that can be placed directly onto the target site. The healing potential of PRP has been shown in several preclinical and clinical studies [13, 16, 17], revealing that its action is directed toward all the articular tissues, ranging from meniscus to cartilage and even soft tissues like synovium, tendons, and ligaments: the effects of PRP are several, including an anabolic stimulus toward cells, an increase in extracellular matrix deposition, reduction of proapoptotic signals, and even an anti-inflammatory effect in the joint environment [13].

In light of such potential, the possibility of applying this biological product to enhance ACL reconstructive surgery appears attractive: PRP not only might promote a better and faster ligamentization of the graft used for ACL reconstruction and reduce the proinflammatory factors released immediately after surgery, but might also contribute to a better integration of the graft within the bone tunnels, thus avoiding their enlargement and failure over time. Furthermore, PRP could be used to accelerate healing and reduce donor-site morbidity at the harvest site of the tendon graft.

The aim of the present paper is to review systematically the current preclinical and clinical evidence concerning the application of PRP as a biological augmentation, to determine safety and efficacy of this biological approach to improve ACL reconstruction surgery.

## 2. Materials and Methods

A systematic review of the literature was performed on the use of PRP in ACL reconstruction. The search was conducted on the PubMed database on February 20th, 2014 using the following parameters: ((ACL) OR (ACL reconstruction) OR (ACL lesion)) AND ((PRP) OR (platelet rich plasma) OR (platelet gel) OR (platelet derived) OR (platelet concentrate)). The guidelines for Preferred Reporting Items for Systematic Reviews and Meta-Analysis (PRISMA) were used. Screening process and analysis were conducted separately by 2 independent observers (BDM and LA).

**Figure 1:**
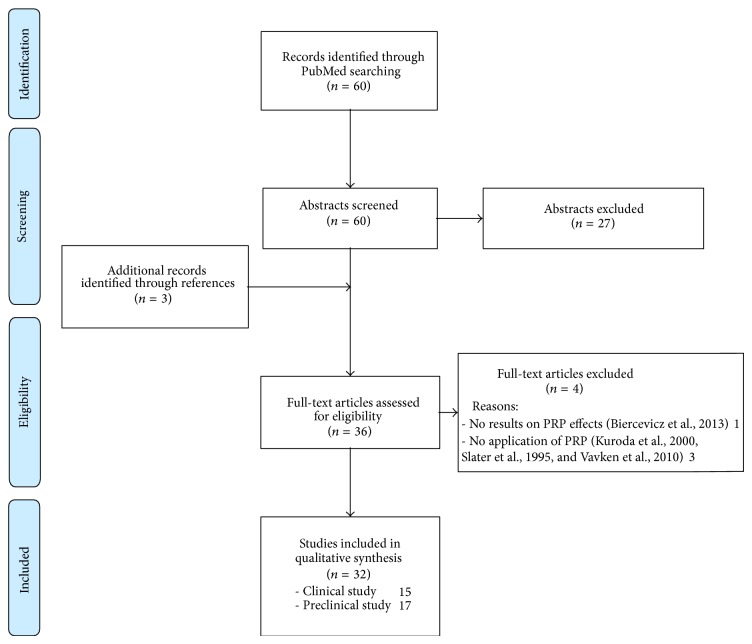
Flowchart of the systematic review.

First, the articles were screened by title and abstract. The following inclusion criteria for relevant articles were used during the initial screening of titles and abstracts: clinical and preclinical reports of any level of evidence, written in English language, with no time limitation, on the use of PRP in ACL reconstruction, and reporting results on PRP effects. Exclusion criteria were articles written in other languages, reviews, or studies analyzing other applications of PRP in knee surgery not related to ACL procedures. In the second step, the full texts of the selected articles were screened, with further exclusions according to the previously described criteria. Moreover, the articles not reporting clinical, MRI, or histologic results were excluded. Reference lists from the selected papers were also screened. A flowchart of the systematic review is provided in Figure 1. Relevant data were then extracted and collected in a unique database with the consensus of the two observers to be analyzed for the purposes of the present paper.

## 3. Results

The database search identified 60 records, and the abstracts were screened and selected according to the inclusion/exclusion criteria. As shown in Figure 1, a total of 33 full-text articles were assessed for eligibility. Four articles [18–21] did not fulfill the criteria and were further excluded, and 3 articles came from the screening of the reference lists, leading to a total of 32 studies included in the final analysis. A detailed description of preclinical studies is reported in Table 1, whereas clinical studies are summarized in Table 2 and discussed in more detail in the following paragraph.

### 3.1. Clinical Evidence

Fifteen clinical trials were selected according to the inclusion criteria: 11 were randomized controlled trials, 3 were prospective comparative studies, and 1 was a retrospective comparative trial (Table 2).

With regard to the graft used to perform ACL reconstruction, in 9 studies, authors used hamstring tendons (gracilis and semitendinosus), in 4 studies bone-patellar tendon-bone (BPB) graft, in 1 study allograft, and in 1 paper authors both hamstring tendons and BPB graft. Fourteen papers described a single-bundle reconstruction technique, whereas just in one trial a double-bundle procedure was used. Concerning PRP delivery methods, in 2 papers (both dealing with BPB graft) PRP was used to promote harvest site healing, in 1 paper PRP was applied intra-articularly by a suprapatellar injection at the very end of the surgical procedure, in 1 paper PRP was used just to coat the intra-articular portion of the graft, and in 11 papers it was administered both on the graft and in the bony tunnels to enhance the graft-bone integration. Among the delivery techniques used, in 2 studies peculiar approaches were applied. While most of the authors applied PRP on the graft surface, Sánchez et al. [22] placed PRP inside the graft through multiple intratendinous depots, while Radice et al. [23] used a more complex technique: before PRP application a bioabsorbable spongy membrane (Gelfoam, Pfizer, New York, NY) was carefully sutured on the harvested tendon and around the femoral bone plug in case of the BPB graft. Then, PRP was administered to allow the spongy membrane to absorb the PRP and avoid its dispersion in the articular space.

In all but one case PRP was activated before administration. Details regarding PRP preparation methods, number of patients included, patients' characteristics, and follow-up evaluations are included in Table 2.

To make the results more understandable, they will be discussed separately according to the specific aspects analyzed in the clinical trials:

- harvest site healing;
- tendon graft maturation and bony tunnel/graft integration;
- clinical results.

### 3.2. Healing of the Harvest Site

Two papers were specifically aimed at assessing the contribution of PRP in the healing of the harvest site after ACL reconstruction with BPB graft, and both of them reported positive outcomes with PRP.

In the study led by de Almeida et al. [24] two groups of patients (15 and 12, resp.) were randomized to receive or not receive PRP after BPB tendon harvesting. Total amount of 20–40 mL of PRP was locally applied and then the peritendineum was sutured. Clinical and MRI evaluations were performed up to 6 months' after operation. The authors found that PRP augmentation determined a significantly smaller patellar tendon gap area with respect to the control group and also a lower postoperation pain reaction in the PRP group. However, at the final clinical evaluation no statistical intergroup difference was recorded in the questionnaires used or in the isokinetic test. In the paper authored by Cervellin et al. [25], 40 patients were included and divided into two treatment groups in the same manner. At 1-year followup, the PRP group showed statistically superior clinical outcomes when evaluated by the VISA-P score and, although not significant, a better bone healing when analyzed by MRI imaging, at both patellar and tibial defect sites (85% of patients in PRP versus 60% in the control group).

### 3.3. Tendon Graft Maturation and Bony Tunnel/Graft Integration

Twelve papers examined outcome regarding (a) graft maturation over time and (b) graft integration in the bone tunnels.

Among the 6 studies reporting data about graft maturation, 4 of them were in favour of PRP augmentation, whereas 2 reported no intergroup difference. With respect to bony tunnels/graft area, 9 studies focused on the integration, documenting in 7 cases no advantage after PRP administration. The evolution over time was investigated more specifically in 3 trials focused on the bony tunnel widening: none demonstrated that PRP was able to prevent tunnels' enlargement over time.

Looking in more detail at the available literature, the first paper was published by Orrego et al. [53] in 2008: 108 patients were divided into 4 treatment groups due to the fact that the authors also used an autologous spongy bone graft (placed by interference fit in the femoral tunnel after graft fixation) as autologous augmentation. A control group was compared to PRP, bone plug, and PRP + bone plug groups. At 6 months MRI revealed a statistically significant advantage for the PRP group in terms of graft maturation (lower signal intensity at MRI) with respect to the control group. However, no differences were observed regarding the evaluation of osteoligamentous interface between the tunnels and the graft and, with regard to tunnel widening, the best results were achieved in the bone plug group which showed the lowest rate of widening, without further advantage from PRP administration. A positive influence of PRP local administration in graft maturation was also shown by other authors. The study authored by Radice et al. [23], who performed ACL reconstruction through both hamstring and PBP grafts, found a better maturation of the intra-articular portion of the graft in the PRP group. In particular, serial postoperation MRI assessments revealed that the mean time to achieve a homogenous “ligamentous-like” signal of the graft was 179 days in PRP group versus 362 days in control group. Subgroup analysis proved that the best responding category was the one where BPB graft reconstruction was performed. Similarly, Sánchez et al. [22] performed macroscopic and histologic evaluations in 37 ACL patients (22 PRP and 15 control) who underwent second-look arthroscopies for other reasons. The authors found that, in patients who received PRP augmentation, there was a superior arthroscopic appearance of the graft (although not significant; *P* = 0.051) and even biopsies revealed a superior tissue quality for PRP, with newly formed synovial-like tissue enveloping the graft in 77% of cases versus 40% of the control group. Seijas et al. [44] were the only authors that administered PRP by an intra-articular injection immediately after closing the arthroscopic portals, thus avoiding its selective delivery onto the graft or into the bony tunnels: the authors found that, despite this unselective administration, there was significant superior graft maturation in PRP group at MRI evaluation both at 3 and 6 months. Finally, with regard to positive PRP effects, Rupreht et al. were the only ones documenting benefit in the bony tunnel/graft area. In a randomized study [47] they showed that PRP administration reduced edema around the tibial tunnel during the first postoperation month and also increased vascular density and microvessel permeability in the proximal tibial tunnel at 1 and 2.5 months' MRI evaluation, thus suggesting that PRP is most effective in the early phases of healing. In another paper [43], the same authors reported that PRP increased cortical bone formation around the tibial tunnel wall at 6 months followup, thus suggesting that PRP might effectively contribute to graft-bone integration.

Among the studies documenting no beneficial effects of PRP administration, the first one was authored by Silva et al. [52] who evaluated the effect of PRP augmentation in promoting graft integration within the femoral tunnels. Forty patients were randomly divided into 4 groups: 10 were controls, 10 received PRP on the graft and in the femoral tunnels, 10 received the same treatment with thrombin-activated PRP, and 10 received further PRP intra-articular injections at 2 and 4 weeks after surgery. MRI controls 3 months after operation showed no statistical intergroup difference regarding maturation of fibrous interzone and, therefore, in osteoligamentous integration. Similar results at 6 months' evaluation were reported by Figueroa et al. [50] who treated 50 patients and analyzed MRI with the purpose of assessing graft maturation and osteoligamentous integration: no significant intergroup difference was found either in the first parameter or in the second. Another study by Vogrin et al. [49] investigated the vascularization at the interface between bone tunnels and graft, and along the intra-articular portion of the graft: a superior vascularization was found in the PRP group only at 4–6 weeks with MRI but not at 10–12 weeks, and no difference was observed with respect to the control group in the vascularization of the intra-articular part of the graft. Another randomized study by Nin et al. [51] on 100 patients revealed that PRP augmentation did not provide superior results in terms of clinical scores, biomechanical tests, and MRI parameters of graft maturation at 12 months evaluation. Furthermore, the authors found no difference either in postoperation swelling or in C-reactive protein levels (inflammatory index) at 10 days after operation Finally, with regard to the evolution of the bony tendon/graft area, beside the previously mentioned study by Orrego et el. [53], more recently Vadalà et al. in 2013 [48] analyzed the role of PRP in preventing bone tunnel enlargement after ACL reconstruction: CT evaluation in 40 patients at a mean of 14.7 months' followup did not reveal any beneficial effect of PRP and furthermore the clinical scores were not positively affected by this biological augmentation. In a similar randomized study on 46 patients led by Mirzatolooei et al. [45] the same findings were reported regarding bone tunnel widening, without any advantage using PRP.

### 3.4. Clinical Results

Seven studies reported clinical outcomes after ACL reconstructive surgery with or without PRP augmentation, focusing on the short-term outcome, with followup reported from 6 months to 2 years. None of them showed any statistical inter-group difference. Only Magnussen et al. in a comparative retrospective trial [46] observed a lower swelling reaction in the PRP group at a mean of 10 days after operation, but the difference was no longer significant after 8 weeks.

## 4. Discussion

The reduction of recovery time and prevention of reinjury after ACL reconstruction are the main goals of sport medicine surgeons. In recent years a number of studies have been published aimed at clarifying the best surgical techniques and considering the different types of grafts, the fixation devices, and other biomechanical and technical variables. Biological augmentation through PRP administration is one of the latest topics for researchers, with the aim of enhancing ACL reconstruction via the help of powerful biological agents.* In vitro* studies and also animal trials have highlighted overall encouraging results (Table 1), thus confirming the expectations about the potential of platelet-derived GFs in stimulating tissue healing: the use of PRP increased the expression of procollagen gene and collagen protein and also contributed to reduce apoptosis and stimulate fibroblast metabolic activity [35–42]. In the animal model it was also observed that PRP was able to determine superior biomechanical properties such as a higher tensile load and linear stiffness of the graft [26–34]. In light of these findings, the application of PRP augmentation in clinical practice appeared justified. However, this systematic review underlines that results are more controversial when looking at clinical published data.

According to the clinical trials currently available, it is possible to highlight some important findings. The first one regards the safety of this approach. The intraoperative use of PRP proved to be safe: in none of the clinical trials considered adverse events related specifically to its use could be identified, and no infections or other complications were reported after PRP administration. PRP actually proved to even reduce the surgical morbidity in two papers [24, 25] specifically investigating its role in promoting graft harvest site healing. In both cases a BPB graft was used for ACL reconstruction and PRP contributed to better healing response evaluated radiographically and, in one case [25], this difference was also reflected in the clinical score (VISA-P). These findings underline that PRP is beneficial in the tendon healing process and confirm the results obtained in other studies dealing with PRP treatment of patellar tendon disease [10]. Based on these results, PRP could be considered as a valid option to address the problem of donor-site morbidity when the patellar tendon is the surgeon's choice for graft harvesting.

More controversial are the results in terms of biological efficacy on the reconstructed ACL. The application of PRP has been hypothesized to improve graft integration within the tunnels. Concerning the osteoligamentous integration, Rupreht et al. showed increased vascular density and microvessel permeability in the proximal tibial tunnel at 1 and 2.5 months' MRI evaluation, as well as reduced bone edema around the tunnel and increased cortical bone formation around the tibial tunnel wall at 6 months followup [43, 47]. Vogrin et al. [49] also showed a significantly higher neovascularization 4–6 weeks after PRP administration, but the advantage of PRP was not confirmed at further followup, thus suggesting that PRP is most effective in the early phases of healing. Other studies did not show any difference in osteoligamentous integration [50, 52], and the three studies, all randomized trials [45, 48, 53] focused on the evolution of bony tunnel/graft area, found that biological augmentation did not contribute to preventing tunnel enlargement with respect to the control group. Therefore, this particular aspect still remains controversial and further studies should better clarify if the role of PRP in bone-graft integration is limited in favouring just the first healing phases.

Also with respect to the graft maturation process, the literature is not univocal, but in this case PRP seems to have a more positive influence. The studies led by Orrego et al., Radice et al., and Seijas et. al proved that PRP augmentation is able to determine a faster and better graft maturation with respect to the control groups [23, 44, 53]. In these trials MRI evaluations revealed that at 4 to 12 months after surgery the signal of the graft in the PRP group was significantly more homogenous and comparable to that of the intact posterior cruciate ligament. Radice et al. [23] reported that PRP augmentation contributed to reducing the time needed to have a homogenous, low-intensity graft signal by almost 50%. Besides MRI findings, Sánchez et al. were able to perform arthroscopic and histological evaluations in a selected group of patients who underwent second-look arthroscopies [22]. The biopsies from PRP-augmented grafts confirmed the superior tissue quality that was hypothesized at imaging. Two studies did not show a significantly better graft maturation following PRP augmentation [50, 51]: however, in both these studies PRP determined superior MRI results and the lack of statistical significance might be attributed to the low sample size included in these trials [54]. Based on this evidence, it is possible to assume that PRP might positively influence graft maturation.

Finally, another important aspect regarding the clinical benefit that could be related to the observed tissue changes in terms of graft integration and maturation. The literature analysis on the clinical outcome of ACL reconstruction did not show any superior results with PRP augmentation. However, the majority of the published clinical studies did not consider clinical results as the primary outcome of the biological augmentation. Clinical evaluations were carried out in only a few studies and were limited to short-term followup, which prevents an accurate analysis of difference in failure rate and revision surgeries between the treatment groups, and further studies at longer followup should focus the real overall clinical benefit of PRP.

The present literature analysis is affected by some limitations. First of all each trial involves a different technique for ACL reconstruction: different types of graft were used, different tunneling, and also different fixation devices, so comparison of results is difficult and, at least hypothetically, it should be considered that some techniques might have a better response to biological stimulation than others. Furthermore, PRP has been administered in different ways: some authors applied it by a simple intra-articular injection after the procedure, others used it to cover the graft before or after its intra-articular fixation, and others injected it into the graft, and PRP could be even combined with substrates to increase ACL augmentation. Moreover, besides the intra-articular area of the graft, some authors also applied PRP inside the bone tunnels: some in both the tibial and femoral one and some only in the femoral one. Therefore, different applicative methods on different specific targets might determine different outcomes. Finally, another confounding factor is directly linked to PRP itself, that is, a well-known and debated topic, that is, the dramatic variability among different PRP formulations [55]. Currently there are several different PRPs in clinical use, differing in terms of preparation methods, activation, and cell content. These features affect the final concentration of GFs and other bioactive molecules delivered* in situ* and might influence the overall regenerative potential of PRP. In light of these aspects, it is even harder to summarize results of clinical trials: each author uses his own formulation and so it is impossible, at present, to establish the best PRP for improving ACL reconstruction, as well as the real potential of this biological approach for ACL augmentation. Considering the great interproduct variability and the various applicative strategies, further high quality studies are needed to determine the best formulation, administration modalities, and the best responding targets of PRP augmentation in ACL reconstructive surgery.

## 5. Conclusion

This systematic review underlines that clinical results on PRP use for ACL augmentation are controversial. The intraoperative use of PRP proved to be safe, and PRP actually showed to even reduce the surgical morbidity promoting graft harvest site healing. Based on current evidence, PRP seems to play a positive role in the healing mechanisms after ACL surgery for what regards graft maturation, whereas the majority of the studies showed no benefit in terms of graft integration, especially in preventing bone tunnel widening. Finally, PRP did not provide a superior clinical outcome at short-term followup, whereas data at longer followup are lacking to address the overall clinical benefit of PRP augmentation.

## Figures and Tables

**Table:** (a) Animal trials

Publication	Animal model	Study protocol	PRP characteristics	Application method	Results
Xie et al., JSR 2013 [26]	36 dogs	PRP versus control	**Platelet count**: 669 ± 51 × 10^9^ pts/L **Activation method**: 10% calcium chloride **Leukocyte count**: —	Injected into ACL graft	PRP allows an increase in expression of vascular endothelial growth factor, thrombospondin-1, neurotrophin-3, growth-associated protein-43, and nerve growth factor mRNA.

Xie et al., JSR 2013 (2) [27]	36 dogs	PRP versus control	**Platelet count**: 669 ± 313 × 10^9^ pts/L **Activation method**: 10% calcium chloride **Leukocyte count**: —	Injected into ACL graft	PRP alters the expression of target genes (growth factor-b1, collagen type1A1, collagen type3A1, decorin, biglycan, matrix metalloproteinase-1, matrix metalloproteinase-13, and tissue inhibitor of metalloproteinase-1 during the remodelling process (evaluations performed at 2, 6, and 12 weeks after surgery).

Mastrangelo et al., JOR 2011 [28]	8 minipigs	Collagen scaffold augmented with PRP 5x versus PRP 3x	**Platelet count**: 1951 ± 304 × 10^9^ pts/L (5x PRP) 1161 ± 179 × 10^9^ pts/L (3x PRP) **Activation method**: — **Leukocyte count**: —	Collagen scaffold augmentation	No statistical difference in biomechanical properties (anteroposterior laxity and structural properties). Histological aspects: compared to 3x PRP, 5x PRP group had significantly greater cellular density and number of vessels and better organization of cells and collagen fibres.

Joshi et al., AJSM 2009 [29]	27 pigs	Collagen-PRP versus control	**Platelet count**: 1279 ± 775 × 10^3^ pts/mm^3^ **Activation method**: — **Leukocyte count**: —	Collagen-PRP composite	The addition of a collagen-PRP composite for ACL repair determined a higher yield load and linear stiffness and higher cell density at 3 months.

Murray et al., JOR 2009 [30]	6 pigs	PRP versus control	**Platelet count**: 2.83 ± 0.53 times **Activation method**: — **Leukocyte count**: 1.95 ± 0.34 times	Injected around the suture material	No significant difference in mechanical properties (ACL laxity, maximum tensile load, and linear stiffness) of repaired ACL.

Murray et al., JOR 2007 [31]	5 pigs	Collagen-PRP versus control	**Platelet count**: 780 − 2300 × 10^3^/mm^3^ **Activation method**: — **Leukocyte count**: —	Collagen scaffold augmentation	Significant improvements in mechanical properties (load at yield, maximum load, and linear stiffness) of repaired ACL compared to sutured only at 4 weeks after surgery.

Murray et al., JOR 2007 (2) [32]	10 dogs	Collagen-PRP versus control	**Platelet count**: — **Activation method**: — **Leukocyte count**: —	Collagen scaffold augmentation	The application of collagen-PRP scaffold allows significant effects of increasing the wound filling and enhancing the presence of fibronectin, fibrinogen, PDGF-A, TBG-b1, FGF-2, procollagen I, and vWF.

Murray et al., JOR 2006 [33]	24 dogs	Collagen-PRP versus control	**Platelet count**: — **Activation method**: — **Leukocyte count**: —	Collagen scaffold augmentation	The application of collagen-PRP scaffold allows significant improvements of histological scores and biomechanical properties, compared to control.

Weiler et al., AJSM 2004 [34]	48 sheep	PRP versus control	**Platelet count**: — **Activation method**: — **Leukocyte count**: —	Injected into ACL graft	Significantly improvements between PRP group and control group concerning cross-sectional area, failure load, stiffness, and tensile stress after 3, 6, or 12 weeks; however, after 24 weeks this difference is not confirmed and mechanical scores are worse than the intact ACL.

**Table:** (b) In vitro trials

Publications	Purpose	PRP characteristics	Application method	Results
Yoshida et al., JOR 2014 [35]	Does increasing platelet concentration enhance ACL fibroblast proliferation and collagen production?	**Platelet count**: PPP: 8 × 10^6^; PRP: 129 × 10^6^ (1x PRP); 370 × 10^6^ (3x PRP); 615 × 10^6^ pts/mL (5x PRP) **Leukocyte count**: 0.03 × 10^6^ cells/mL	Scaffold's augmentation (collagen scaffold)	Highest cell metabolism, lowest apoptosis rates, and highest collagen gene expression with PPP and 1x PRP.

Yoshida and Murray, JOR 2013 [36]	How peripheral blood mononuclear cells (PBMCs) in PRP affect fibroblast behaviour?	**Platelet count**: PRP: 911 × 10^6^ pts/mL **Leukocyte count**: PRP: — PBMC: 5 × 10^6^ cells/mL	Scaffold's augmentation (collagen scaffold)	PRP with PBMCs determined an increasing of type I and type III procollagen gene expression, collagen protein expression, and cell proliferation. An increase of IL-6 expression was detected in PBMCs exposed to PRP. No effect of PBMCs without PRP.

Cheng et al., JOR 2012 [37]	Does cell's age influence the ACL cell response to PRP?	**Platelet count**: 628 × 10^6^ pts/mL **Leukocyte count**: —	Scaffold's augmentation (collagen scaffold)	PRP increases cellular metabolic activity and reduced apoptotic rate and stimulation of collagen production on immature and adolescent cells, compared to collagen only scaffold. Lower effects of PRP on adult cells.

Fallouh et al., JBJSAm 2010 [38]	Effects of autologous PRP on cell viability and collagen synthesis of human ACL cells.	**Platelet count**: — **Activation method**: 10% thrombin solution **Leukocyte count**: —	PRP clot added to culture environment	PRP group had higher concentration of growth factors, cell number, and total collagen production, compared to PPP group.

Magarian et al., Knee 2011 [39]	Age dependence of ACL fibroblast response to PRP	**Platelet count**: 684 × 10^9^ pts/L **Leukocyte count**: 2.78 × 10^9^ cells/L	Scaffold's augmentation (collagen scaffold)	The comparison between immature and adolescent cells showed a significantly higher cell migration and proliferation in immature group, whereas no differences were seen in scaffold contraction.

Cheng et al., TissEngA 2010 [40]	Does PRP components (pts or PPP) independently influence ACL cells behaviour?	**Platelet count**: 911 × 10^6^ pts/mL **Leukocyte count**: —	Scaffold's augmentation (collagen scaffold) with PRP, pts, or PPP	The addition of PPP, platelets, or PRP all reduced cell apoptosis and enhanced metabolic activity of fibroblasts. Significantly higher expression of collagen only with PRP.

Mastrangelo et al., JOR 2010 [41]	Age dependence in ACL healing	**Platelet count**: 915 × 10^9^ pts/L (porcine PRP); 369 × 10^9^ pts/L (ovine PRP) **Leukocyte count**: 5.37 × 10^9^ cells/L (porcine PRP); 9.6 × 10^9^ cells/L (ovine PRP)	Scaffold's augmentation (collagen scaffold)	Comparison between adult, adolescent, and immature cells concerning cell proliferation and cellular migration: better results for immature cells.

Scherping et al., CTR 1997 [42]	Analysis of effects of single growth factors (GF) on fibroblasts harvested from medial collateral ligament and ACL of skeletally mature rabbits	**Growth factor analysed are as follows**: epidermal GF, basic fibroblast GF, platelet derived GF-BB, acid fibroblast GF, TGF-b1, insulin-like GF-1, platelet derived GF-AA, and interleukin-1a		Epidermal GF, basic fibroblast GF, and platelet derived GF-BB determined a significantly higher fibroblast's proliferation than the untreated cells. No significant difference reported for the others GFs analysed.

**Table 2:** Synopsis of the clinical studies dealing with the use of PRP in ACL reconstruction.

Publication	Study protocol	Purpose	Patients characteristic	PRP characteristics	ACL reconstruction technique	Application method	F-Up	Results
Rupreht et al., RadioOncol 2013 [43]	Randomized trial (PRP versus control)	MRI quantitative evaluation of *tunnel wall cortical bone (TCB) formation *	41 (21 versus 20) **Age**: 37.2 versus 32.6 **Sex**: 13 M—8 F versus 15 M—5 F	**Preparation method**: — **Platelet count**: — **Activation method**: — **Leukocyte**: —	Double-looped semitendinosus and gracilis tendon autograft. Fixation: 2 bioabsorbable cross-pins in the femoral tunnel and one bioabsorbable interference screw in the tibial tunnel.	After autograft positioning into the femoral and tibial tunnels (1 mL in each of them), and onto the graft itself (3 mL) without arthroscopic fluid.	6 m	A gradual increase in the percentage of the tunnel wall consisting of tunnel wall cortical bone (TCB) during the followup was observed. At six months the mean percentage of TCB was significantly higher (*P* = 0.003) in the PRP group than in the control group.

Seijas et al., JOR 2013 [44]	Randomized trial (PRP versus control)	MRI evaluation of *remodelling stages of the graft *	98 (49 versus 49) **Age**: — **Sex**: —	**Preparation method**: PRGF technique (BTI Systems Vitoria, Spain) **Platelet count**: — **Activation method**: calcium chloride **Leukocyte**: N?	Autologous patellar tendon grafts with bone plugs of 9 mm. Fixation: hydroxylapatite screws in the femur and tibia.	8 mL of PRP percutaneously injected into the suprapatellar joint after portal suture.	12 m	More patients in the PRP group than controls attained higher stages of remodelling at month 4 (*P* = 0.003), month 6 (*P* = 0.0001), and month 12 (but NS *P* = 0.354).

Mirzatolooei et al., BJJ 2013 [45]	Randomized trial (PRP versus control)	Clinical, CT, and arthrometric evaluation of PRP role in *prevention of tunnel widening* after ACL reconstruction	46 (23 versus 23) **Age**: 26.4 versus 26.9 **Sex**: 20 M—3 F versus 22 M—1 F	**Preparation method**: Double syringe system (Arthrex) **Platelet count**: — **Activation method**: none **Leukocyte**: few	Single-bundle quadrupled autograft of hamstrings. Fixation: a cross-pin in the femoral tunnel and a bioabsorbable interference screw in the tibial tunnel	Graft immersed in the PRP solution for five minutes before implantation; 2 mL of PRP injected into the femoral tunnel and 1.5 mL into the tibial tunnel at the end of the surgery.	3 m	Despite slightly less tunnel widening in the PRP group, there were no significant differences at any of the sites of measurement between immediately after surgery and three months postoperatively.

Magnussen et al., Knee 2013 [46]	Retrospective comparative study (PRP versus control)	Evaluation of the effect of intraoperative PRP on patient-reported *clinical outcomes *	58 (29 versus 29) **Age**: 35.1 versus 35.3 **Sex**: —	**Preparation method**: GPS II Platelet Concentrate Separation Kit (Biomet, Inc., Warsaw, IN, USA) **Platelet count**: — **Activation method**: calcium chloride **Leukocyte**: Y?	Allograft tibial tendon. Fixation: an absorbable cross-pin in the femoral tunnel and an absorbable interference screw in the tibial tunnel	After graft positioning intra-articular portion of the graft was coated with PRP.	24 m	Decreased effusions at 10 ± 4 days were noted in the PRP group, but this difference disappeared by 8 ± 4 weeks. No differences in patient-reported outcomes were noted in the 58 patients with two-year outcome data.

Rupreht et al., JMRI 2013 [47]	Randomized trial (PRP versus control)	Evaluate if PRPG has an influence on the extent of the *edema and vascularity in the tibial tunnel* that can be assessed by DWI and DCE-MRI	41 (21 versus 20) **Age**: 37.2 versus 32.6 **Sex**: 13 M—8 F versus 15 M—5 F	**Preparation method**: — **Platelet count**: 190 × 10^9^/L **Activation method**: — **Leukocyte**: —	Double-looped semitendinosus and gracilis tendon autograft. Fixation: 2 bioabsorbable cross-pins in the femoral tunnel and one bioabsorbable interference screw in the tibial tunnel.	Applied after autograft positioning, into the femoral and tibial tunnels (1 mL in each of them), as well as onto the graft itself (3 mL) without arthroscopic fluid	6 m	DWI and DCE-MRI measurements indicate a reduced extent of edema during the first postoperative month as well as an increased vascular density and microvessel permeability in the proximal tibial tunnel at 1 and 2.5 postoperative months as the effect of the application of PRPG.

Vadalà et al., KSSTA 2013 [48]	Randomized trial (PRP versus control)	CT evaluation of the efficacy of platelet-rich plasma (PRP) in reducing *femoral and tibial tunnel enlargement and clinical score *	40 (20 versus 20) **Age**: 34.5 **Sex**: 40 M	**Preparation method**: PRP Fast Biotech kit (MyCells PPT-Platelet Preparation Tube). **Platelet count**: — **Activation method**: addition of thrombin and 10% Ca-gluconate **Leukocyte**: Y?	ACL reconstruction with hamstrings (Out-In technique). Fixation: Swing-Bridge device on the femoral side and Evolgate screw on the tibial side.	(i) 5 mL of PRP between the peripheral part of the graft and the femoral tunnel wall; (ii) 5 mL of PRP in its semisolid pattern above the graft; (iii) 5 mL of liquid and semisolid PRP on the tibial side.	14.7 m	The use of PRP does not seem to be effective in preventing tunnel enlargement. Physical examination as well as the evaluation scales used showed no differences between the two groups.

de Almeida et al., AJSM 2012 [24]	Randomized trial (PRP versus control)	MRI evaluation of *healing of patellar tendon harvest site *	27 (12 versus 15) **Age**: 25.8 versus 23.1 **Sex**: 10 M—2 F versus 14 M—1 F	**Preparation method**: Haemonetics MCS1 9000 cell separator with a specific kit for platelet apheresis 995-E (Haemonetics Corp, Braintree, Massachusetts) **Platelet count**: 1,185,166/mm^3^ ± 404,472/mm^3^ **Activation method**: thrombin and 0.8 mL of calcium chloride **Leukocyte**: 0.91/mm^3^ ± 0.81/mm^3^	Autologous bone-patellar tendon-bone graft. Fixation: absorbable transverse double pin system in the femur and an absorbable interference screw in the tibia.	The patellar tendon defect was completely filled with 20 to 40 mL of PRP gel and the peritendon was closed with absorbable 3–0 sutures.	6 m	Patellar tendon gap area was significantly smaller (*P* = 0.046) in the PRP group (4.9 ± 5.3 mm^2^) than in the control group (9.4 ± 4.4 mm^2^). Visual analog scale score for pain was lower in the PRP group immediately postoperatively (3.8 ± 1.0) than in the control group (5.1 ± 1.4). There were no differences after 6 months in questionnaire and isokinetic testing results comparing both groups.

Cervellin et al., KSSTA 2012 [25]	Randomized trial (PRP versus control)	MRI and clinical evaluation of *healing of patellar tendon harvest site *	40 (20 versus 20) **Age**: 22.9 versus 22.7 **Sex**: 40 M	**Preparation method**: the Gravitational Platelet Separation II (GPS) system (Biomet Biologics, Inc., Warsaw, IN, USA) **Platelet count**: — **Activation method**: thrombin and calcium chloride **Leukocyte**: Y?	Autologous bone-patellar tendon-bone graft. Fixation: —	PRP was applied to both the patellar and tendon-bone plug harvest site and stabilized by peritendon suture	12 m	VISA scores were significantly higher in the patients treated with PRP, whereas no significant difference in postoperative VAS scores between the two groups was observed. In 85% of PRP group patients, the tibial and patellar bone defect was satisfactorily filled by new bony tissue, whereas this percentage was just of 60% in control group patients, but this difference was not statistically significant.

Vogrin et al., ESR 2010 [49]	Randomized trial (PRP versus control)	MRI evaluation of the *revascularization process* in the osteoligamentous interface zone in the bone tunnels and in the intra-articular part of the graft after ACL reconstruction	41 (21 versus 20) **Age**: 37.2 versus 32.6 **Sex**: 13 M—8 F versus 15 M—5 F	**Preparation method**: Magellan (Medtronic Biologic Therapeutics and Diagnostics, Minneapolis, MN, USA) **Platelet count**: 160 − 350 × 10^3^/*µ*L **Activation method**: autologous human thrombin **Leukocyte**: present (?)	Double-looped semitendinosus and gracilis tendon graft. Fixation: 2 bioabsorbable cross-pins in the femoral tunnel and 1 bioabsorbable interference screw in the tibial tunnel.	PRP was applied into the femoral and tibial tunnels as well as onto the graft itself.	4–6 weeks	After 4–6 weeks, the PRP-treated group demonstrated a significantly higher level of vascularization in the osteoligamentous interface (0.33 ± 0.09) than in the control group (0.16 ± 0.09; *P* < 0.001). In the intra-articular part of the graft, we found no evidence of revascularization in either group.

Figueroa et al., Arthroscopy 2010 [50]	Comparative study (PRP versus control)	MRI evaluation of *integration and maturation* of semitendinosus-gracilis (STG) grafts in anterior cruciate ligament (ACL)	50 (30 versus 20) **Age**: 26.8 versus 23.6 **Sex**: 18 M—12 F versus 15 M—5 F	**Preparation method**: Magellan system (Medtronic, Minneapolis, MN) **Platelet count**: — **Activation method**: autologous human thrombin **Leukocyte**: Y	ACL reconstruction with hamstring tendons (ST-G). Fixation: a cross-pin in the femoral tunnel and a bioabsorbable interference screw in the tibial tunnel.	PRP was applied under arthroscopy in both the tibial (3 mL) and femoral (3 mL) tunnels with a long needle syringe and directly applied in the intra-articular graft portion (4 mL)	6 m	No statistically significant benefit in the PRP group in terms of integration assessment and graft maturation (ligamentization).

Sánchez et al., Arthroscopy 2010[22]	Comparative study (PRP versus control)	Macroscopic and histologic evaluation of *ligamentization* of tendon grafts	37 (22 versus 15) **Age**: 28 **Sex**: 26 M—11 F	**Preparation method**: BTI System II (BTI Biotechnology Institute, Vitoria, Spain) **Platelet count**: 2- to 3-fold the platelet count of peripheral blood **Activation method**: calcium chloride **Leukocyte**: scarce leukocytes	ACL reconstruction with hamstring tendons. Fixation: transcondylar screw proximally and PRGF-treated bone plug and 2 metal staples distally.	Six mL PRP was injected within the tendon graft fascicles with several punctures performed along the graft length, graft soaked in PRP until implantation and the remaining aliquots were applied at the portals during suturing.	15 m	Overall, arthroscopic evaluations were not statistically different between PRGF and control groups (*P* = 0.051). PRGF treatment influenced the histologic characteristics of the tendon graft, resulting in tissue that was more mature than in controls (*P* = 0.024). Histologically evident newly formed connective tissue enveloping the graft was present in 77.3% of PRGF-treated grafts and 40% of controls.

Radice et al., Arthroscopy 2010 [23]	Comparative study (PRP versus control)	MRI evaluation of PRPG effect on *cell proliferation and collagen production* in the human tendon and plays a key role in the remodeling and repair processes of the graft used in ACL reconstruction.	50 (25 versus 25) **Age**: 30 versus 32 **Sex**: 18 M—7 F versus 21 M—4 F	**Preparation method**: GPS system of Biomet (Warsaw, IN) **Platelet count**: — **Activation method**: calcium chloride **Leukocyte**: Y?	BPTB autograft (15 versus 10) or hamstring (10 versus 15). Fixation: in BPTB autograft metallic interference screws; in hamstring autograft metallic or bioabsorbable cross-pin in the distal femur; and a bioabsorbable screw with a metallic staple in the proximal tibia.	PRP administered with the help of a sutured and compressed Gelfoam; 5 mL PRP was added homogeneously so as to completely cover the graft.	9 versus 12 m	ACL reconstruction with the use of PRPG achieves complete homogeneous grafts assessed by MRI, in 179 days compared with 369 days for ACL reconstruction without PRPG. This represents a time shortening of 48% with respect to ACL reconstruction without PRPG.

Valentí Nin et al., 2009 Arthroscopy [51]	Randomized trial (PRP versus control)	To evaluate and compare the *clinical and inflammatory* parameters with the addition of platelet-derived growth factor (PDGF) in primary anterior cruciate ligament (ACL) reconstruction with bone-patellar tendon-bone allograft.	100 (50 versus 50) **Age**: 26.1 versus 26.6 **Sex**: 40 M—10 F versus 38 M—12 F	**Preparation method**: 40 mL of citrated blood was centrifuged for 8 minutes at 3,000 rpm (2,217 g) by use of a standard centrifuge **Platelet count**: 837 × 10^3^/mm^3^ **Activation method**: 10% calcium chloride **Leukocyte**: present (?)	ACL reconstruction with patellar tendon allograft. Fixation: 2 biodegradable cross-pins in the femoral bone and a tibial biodegradable interference screw.	Ligament covered with PRP and sutured over itself with PRP in its interior. The rest of the gel was introduced after implantation of the graft inside the tibial tunnel.	18 m	The results did not show any statistically significant differences between the groups for inflammatory parameters, magnetic resonance imaging appearance of the graft, and clinical evaluation scores.

Silva and Sampaio, KSSTA 2009 [52]	Randomized trial (4 groups: group A control; group B PRP in FT; group C with PRP in FT and intra-articular at 2 and 4 weeks; group D with PRP activated with thrombin in FT)	To assess with magnetic resonance (MR) imaging if the PRP *accelerates tendon-to-bone integration in the femoral tunnel* (*FT*) after hamstring double-bundle ACL reconstruction.	40 (10 versus 10 versus 10 versus 10) **Age**: 26.8 **Sex**: 38 M—2 F	**Preparation method**: Mini GPS III Kit (Biomet) **Platelet count**: — **Activation method**: calcium chloride **Leukocyte**: Y?	Double-bundle arthroscopic ACL reconstruction with autologous hamstring tendons. Fixation: 2 Endobutton for the AM and PL bundle in the femur, 2 bioabsorbable interference screw in the tibia.	PRP was placed between the strands of the graft in each femoral tunnel.	3 m	The graft integration is not complete at 3 months after surgery in the PL and AM femoral tunnel, using Endobutton CL for fixation, and the use of PRP isolated or with thrombin seems not to accelerate tendon integration

Orrego et al., Arthroscopy 2008 [53]	Randomized trial (lesser quality, 4 groups: control, PC, BP, and PC + BP)	Determine if the use of platelet concentrate (PC) and bone plug (BP) does accelerate the healing process in anterior cruciate ligament (ACL) reconstruction, in terms of *maturation of the graft, osteoligamentous interface, and widening of the femoral tunnel. *	108 (27 versus 26 versus 28 versus 27) **Age**: 30 **Sex**: 99 M—17 F (−8 dropout)	**Preparation method**: Biomet GPS II kit (Biomet, Warsaw, IN) **Platelet count**: — **Activation method**: calcium chloride **Leukocyte**: Y?	ACL reconstruction with quadruple STG. Fixation: a biodegradable transfixing pin proximally and a biodegradable interference screw distally; the bone plug was placed by interference fit at the level of the femoral tunnel.	Five mL PRP was added between the strands of the quadruple STG graft before passing into the tunnel. After fixation, 1 mL of PRP was injected into the femoral tunnel between the strands of the graft.	6 m	The use of PC had an enhancing effect on the graft maturation process evaluated only by MRI signal intensity, without showing any significant effect in the osteoligamentous interface or tunnel widening evolution. The use of a BP effectively prevented tunnel widening. The BP and PC combination did not show a synergic effect as compared to PC or BP individually.
